# Assessing the perioperative outcomes of abdominal drain omission after robot-assisted partial nephrectomy

**DOI:** 10.1038/s41598-024-59404-w

**Published:** 2024-04-15

**Authors:** Francesco Ditonno, Riccardo Bertolo, Alessandro Veccia, Sonia Costantino, Francesca Montanaro, Francesco Artoni, Alberto Baielli, Michele Boldini, Davide Brusa, Vincenzo De Marco, Filippo Migliorini, Antonio Benito Porcaro, Riccardo Rizzetto, Maria Angela Cerruto, Riccardo Autorino, Alessandro Antonelli

**Affiliations:** 1 Department of Urology, Borgo Trento Hospital, University of Verona, Azienda Ospedaliera Universitaria Integrata Verona, AUOI Verona, Piazzale Aristide Stefani 1, 37126 Verona, Italy; 2 Department of Urology, Rush University Medical Center, Chicago, IL USA

**Keywords:** Renal neoplasm, Robotic, Nephrectomy, Drain, Complications, Renal cancer, Surgical oncology

## Abstract

The study aimed to evaluate the impact of abdominal drain placement (vs. omission) on perioperative outcomes of robot-assisted partial nephrectomy (RAPN), focusing on complications, time to canalization, deambulation, and pain management. A prospectively-maintained institutional database was queried to get data of patients who underwent RAPN for renal masses between January 2018 and May 2023 at our Institution. Baseline, surgical, and postoperative data were collected. Retrieved patients were stratified based upon placement of abdominal drain (Y/N). Descriptive analyses comparing the two groups were conducted as appropriate. After adjusting for potential confounders, a logistic regression analysis was conducted to evaluate significant predictors of any grade and “major” complications. 342 patients were included: 192 patients in the “drain group” versus 150 patients in the “no-drain” group. Renal masses were larger (*p* < 0.001) and at higher complexity (RENAL score, *p* = 0.01), in the drain group. Procedures in the drain group had statistically significantly longer operative time, ischemia time, and higher blood loss (all *p*-values < 0.001). The urinary collecting system was more likely involved compared to the no-drain group (*p* = 0.01). At multivariate analysis, abdominal drainage was not a significant predictor of any grade (OR 0.79, 95%CI 0.33–1.87) and major postoperative complications (OR 3.62, 95%CI 0.53–9.68). Patients in the drain group experienced a statistically significantly higher hemoglobin drop (*p* < 0.01). Moreover, they exhibited statistically significant higher paracetamol consumption (*p* < 0.001) and need for additional opioids (*p* = 0.02). In summary, the study results suggest the safety of omitting drain placement and remark on the need for personalized decision-making, which considers patient and procedural factors.

## Introduction

The treatment paradigm of renal cell carcinoma has evolved over time, towards increasingly minimally-invasive and conservative approaches, with robot-assisted partial nephrectomy (RAPN) emerging as its pinnacle.

The prophylactic placement of an abdominal drain as the conclusive act of a major surgical intervention represents a common practice in urologic surgery due to its diagnostic and therapeutic role, enabling early detection of bleeding events and prevention of fluid collection^1^.

The issue arises due to the relatively low incidence of such complications, even in cases of RAPN for large renal masses or imperative indications, which raises doubts about the appropriateness of drain placement^2,3^. Furthermore, it remains uncertain whether the placement of an abdominal drain effectively reduces the likelihood of surgical complications and hospital readmission^4^. Moreover, discomfort and postoperative pain have been reported in relation to the placement of a drain after abdominal surgery^5^.

In such an uncertain scenario, the present analysis aimed to evaluate the impact of abdominal drain placement on the perioperative surgical outcomes of patients undergoing RAPN, focusing on patient-related outcomes such as time to canalization, deambulation, and postoperative pain management.

## Materials and methods

### Patient selection

A prospectively maintained institutional database relative to patients undergoing kidney surgery for renal masses was queried to retrieve data of consecutive patients who underwent RAPN for renal masses between January 2018 and May 2023 at our Institution. Patients with a history of prior renal surgery, and/or multiple ipsilateral or bilateral tumors, and/or patients with tumor(s) diagnosed in solitary or horseshoe kidneys were excluded. Additionally, patients with missing data regarding the outcomes of interest and/or incomplete follow-up data were excluded. All procedures were conducted in accordance with ethical standards and the principles of the Declaration of Helsinki. The local ethics committee (Comitato etico Territoriale Area Sud-Ovest Veneto—CET-ASOV) did not consider the application on the present clinical audit/evaluation of aggregate/anonymous data from the institutional routinely gathered record.

### Surgical technique

Experienced robotic surgeons (number of robotic surgeries completed before the study start > 150) performed all the procedures using the da Vinci Surgical System^6^. A standardized surgical approach, including hilar dissection, renal lesion preparation, and intraoperative ultrasonography, was adopted for all cases. Of interest for the present study is that the decision to place an abdominal drain at the end of the surgery was based upon the surgeon’s preference.

### Variables and outcomes

Baseline characteristics, surgical, and postoperative data were collected. The analyzed variables included:

- Baseline features: demographic data, body mass index (BMI), Charlson comorbidity index (CCI)^7^, preoperative serum creatinine (SCr) and estimated glomerular filtration rate (eGFR) calculated by using the Chronic Kidney Disease Epidemiology Collaboration formula^8^, tumor size, and RENAL nephrometry score^9^.
- Intraoperative features: type of clamp, warm ischemia time, operative time, urinary system involvement and repair, renorrhaphy technique (single-vs. double-layer) and eventual use of bolstering and/or hemostatic agents, estimated blood loss, and the occurrence of intraoperative complications.
- Postoperative features: type and grade of postoperative complications according to Clavien-Dindo [CD] classification^7^ (complications with CD grade ≥ III were defined as “major complications”), length of stay, re-admission rate within 90 days, pathology data (histology, pT stage, tumor grading, and margin status), and blood test outcomes (hemoglobin [Hb], SCr and eGFR at post-operative day I, discharge and last follow-up).

Data regarding mobilization with deambulation, return to bowel function (referred to as the resumption of complete bowel function, including the passage of flatus and stool), evaluation of perceived pain at discharge (assessed by the visual analogue scale—VAS), and the pain management strategy (grams of paracetamol prescribed during hospitalization, and/or eventual need for additional non-steroidal anti-inflammatory drugs—NSAIDs—and/or use of opioids for analgesia) were retrieved for the purpose of the study. Late deambulation was defined as a time to deambulation > 3 days, and late canalization as a time to canalization > 2 days.

In addition, Hb drop, defined as the difference between baseline and discharge values, and eGFR variation, defined as the difference between baseline and last follow-up values were calculated.

Finally, the temporal trend of abdominal drain placement was assessed.

### Statistical analysis

Statistical analysis was conducted following published guidelines^10^. The cohort was divided into two groups based upon the placement (or not) of an abdominal drain. Median (IQR) was adopted to report continuous variables. Proportion and frequencies were used to report categorical variables. Descriptive analyses comparing the two groups were conducted using the Mann Whitney U-test for continuous variables, and the Fisher's exact test for categorical variables. A logistic regression analysis evaluated significant predictors of any grade and “major” complications after adjusting for potential confounders. After dividing the study period into 2-year timeframes, the Cochran-Armitage trends test was applied to analyze the change over time in the proportion of tubeless (i.e., performed without the placement of a drain) procedures as a fraction of the total procedures performed during the study period. Stata® 17.0 (StataCorp LLC, College Station, TX, USA) was used for statistical analysis with statistical significance set at *p* ≤ 0.05.

### Ethical approval

Institutional and/or licensing committee approval was not obtained due to the retrospective nature of the present analysis (ethical approval waiver by Comitato etico Territoriale Area Sud-Ovest Veneto—CET-ASOV, Azienda Ospedaliera Universitaria Integrata Verona c/o UOC Farmacia, P.le Stefani, 1 37,126 Verona).

### Informed consent

Informed consent was obtained from all subjects and/or their legal guardian(s).

## Results

### Baseline features

A total of 342 patients underwent RAPN for renal masses at our institution during the study period and met the inclusion criteria. Of these, 192 patients had abdominal drains placed at the end of the surgery, while 150 patients did not. The groups had comparable baseline characteristics, except for a statistically significantly higher proportion of males in the no-drain group. Moreover, renal masses were larger (*p* < 0.001) and of higher complexity according to RENAL score (*p* = 0.01) in the drain group (Table 1).

**Table 1:** Baseline features.

	No drain	Drain	*p*
	(150)	(192)	
Age, years			
Median (IQR)	62 (54–71)	64 (55–73)	.1
Sex, no. (%)			
Male	115 (76.7)	124 (64.6)	**.01**
BMI			
Median (IQR)	26.8 (23.9–29.1)	26 (23.9–29)	.5
CCI			
Median (IQR)	2 (2–3)	2 (2–3)	.9
ASA, no. (%)			
≥ 3	38 (25)	50 (26)	.9
History of abdominal surgery, no. (%)	13 (8.7)	20 (10.4)	.7
Preoperative Cr			
Median (IQR)	0.92 (0.81–1.07)	0.90 (0.78–1.08)	.9
Preoperative eGFR			
Median (IQR)	85.7 (72–97.5)	86 (72.4–95)	.4
Diameter, cm			
Median (IQR)	2.7 (2–3.8)	3.2 (2.5–5)	** < .001**
Location, no. (%)			
Hilar	7/147 (4.8)	17/184 (9.2)	.1
Anterior	71/145 (49)	79/177 (44.6)	.5
Polar	79/142 (55.6)	93/158 (58.9)	.8
Nephrometry score			
RENAL, median (IQR)	7 (5–8)	7 (6–9)	**.01**

ASA (American Society of Anesthesiology) BMI (Body mass index); CCI (Charlson Comorbidity Index); Cr (Serum creatinine); eGFR (estimated glomerular filtration rate).

Student’s T test was used for continuous variables.

Fisher's exact test was used for categorical variables.

Significant values are in bold.

### Surgical outcomes

Procedures in the drain group had statistically significantly longer operative (163 min [130–204] vs. 128 min [103–157]) and ischemia time (19 min [14–25] vs. 13 min [8–18]), with higher blood loss (200 mL [87.5–300] vs. 50 mL [0–150]) (all *p*-values < 0.001). The on-clamp approach was more likely adopted in the drain group (64.4% vs. 52.7%, *p* = 0.03). The same was true for single-layer renorrhaphy (66.1% vs. 61.7%, *p* = 0.03). More patients in the drain group had a statistically significantly higher violation of the urinary collecting system (6.3% vs. 0.7%, *p* = 0.01), with dedicated closure of the urinary tract performed in 5.3% and 0.7%, respectively (*p* = 0.02). Conversely, there was no difference in the utilization of bolstering (*p* = 0.1) and hemostatic agents (*p* = 0.6) to achieve intraoperative hemostasis between the two groups (Table 2).

**Table 2:** Surgical outcomes.

	No drain	Drain	*p*
	(150)	(192)	
Approach, no. (%)			
Transperitoneal	142 (94.7)	171 (89.1)	.07
WIT, min			
median (IQR)	13 (8–18)	19 (14–25)	** < .001**
EBL, mL			
median (IQR)	50 (0–150)	200 (87.5–300)	** < .001**
OT, min			
median (IQR)	128 (103–157)	163 (130–204)	** < .001**
Clamp, no. (%)			
Yes	78/148 (52.7)	123/191 (64.4)	**.03**
Renorrhaphy, no. (%)			
Yes	144/149 (96.6)	190 (100)	**.01**
Single Layer	84/136 (61.7)	113/171 (66.1)	**.03**
Urinary system involvement, no. (%)			
Yes	1/148 (0.7)	12 (6.3)	**.01**
Closure	1/148 (0.7)	10 (5.3)	**.02**
Hemostasis, no. (%)			
Bolstering	83/124 (66.9)	132/177 (74.6)	.1
Hemostatic agent	132 (88)	173 (90.1)	.6
Intraoperative complication, no. (%)	2 (1.3)	3 (1.6)	1

EBL, estimated blood loss; OT, operative time; WIT, warm ischemia time.

Student’s T test was used for continuous variables.

Fisher's exact test was used for categorical variables.

Significant values are in bold.

### Postoperative outcomes

**Figure 1:**
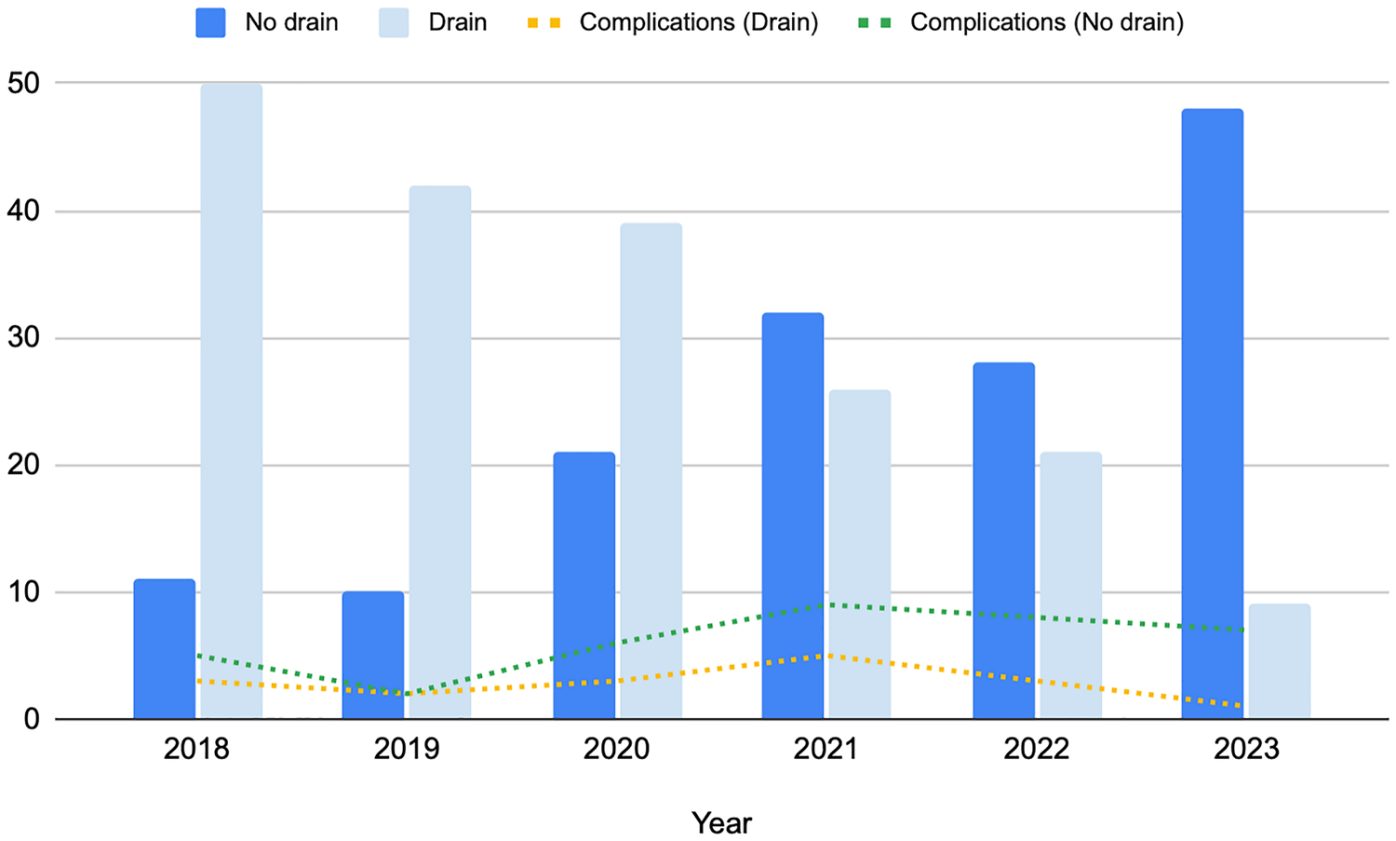
Bar chart showing the number of patients who had a drain placed versus those who did not. The dotted lines represent the respective occurrences of postoperative complications throughout the study period.

Within the drain cohort, the median (IQR) time to drain removal was 2 days (1–4). Patients in the drain group exhibited a statistically significantly longer length of stay (*p* < 0.001). The incidence of any-grade postoperative complications was not statistically different (*p* = 0.05), but major complications were significantly higher in the drain group (4.7%) compared to the no-drain group (0.6%, *p* = 0.04) (Table 3). Hemoglobin levels were statistically significantly lower in the drain group on post-operative day I and at discharge, with a statistically significantly higher Hb drop compared to the no-drain group (all *p*-values < 0.01). Regarding the management of complications, no difference in bleeding events was observed (*p* = 0.1); however, the drain group had a significantly higher rate of postoperative transfusion (5.7% vs. 0%, *p* < 0.01). Moreover, the two cohorts were comparable in terms of number of patients experiencing late canalization (drain: 9% vs. no-drain: 7.4%, *p* = 0.6) and deambulation (drain: 2.7% vs. no-drain: 3.8%, *p* = 0.7). While pain at discharge was similar between patients with and without an abdominal drain (*p* = 0.3), the former cohort exhibited a statistically significant increase in the need for analgesics, both in terms of grams of paracetamol (*p* < 0.001) and need for additional opioids (*p* = 0.02), with no significant difference in the prescription of additional NSAIDs (*p* = 0.09). At multivariate analysis (Table 4), the placement of an abdominal drain was found to be neither a significant predictor of overall complications (OR 0.79, 95% CI 0.33–1.87) nor major postoperative complications (OR 3.62, 95% CI 0.53–9.68). When compared to the total number of procedures performed in each timeframe, the rate of tubeless procedures was 19% for 2018–2019 versus 45% for 2020–2021 versus 72% for 2022–2023, reflecting a statistically significant rising trend with slope (of regression line) = 0.265, *p* < 0.001 (Fig. 1).

**Table 3:** Postoperative outcomes.

	No drain	Drain	*p*
	(150)	(192)	
Pathology, no. (%)			
ccRCC	79/132 (59.8)	122/179 (68.2)	.08
≥ pT2	5/131 (3.8)	15/176 (8.5)	.1
Fuhrman ≥ 3	22 (14.7)	31 (16.1)	.6
Positive margin	10/140 (7.1)	15/181 (8.3)	.8
Postop complication, no. (%)			
Yes	17 (11.3)	37/191 (19.4)	.05
CD ≥ 3	1 (0.6)	9/191 (4.7)	**.04**
Urine leak	0 (0)	2 (1)	0.5
Bleeding	13 (8.7)	26 (13.5)	.1
Transfusion	0 (0)	11 (5.7)	** < .01**
Embolization	0 (0)	4 (2.1)	.1
Ureteral stenting	0 (0%)	1 (0.5%)	1
I POD, median (IQR)			
Hb	13.4 (12–14.2)	12.7 (11.5–13.7)	** < .001**
Cr	1.01 (0.89–1.18)	1.01 (0.85–1.3)	.8
eGFR	81 (61–91)	75.8 (57.2–88)	.1
Discharge, median (IQR)			
Hb	13.1 (11.7–13.8)	12.4 (11.3–13.5)	** < .001**
Cr	0.94 (0.83–1.14)	0.94 (0.79–1.18)	.9
eGFR	84 (65.5–93.2)	82 (61–93.5)	.2
Hb drop, median (IQR)^§^	− 1.5 (− 2.2, − 0.9)	− 1.9 (− 2.6, − 1.1)	** < .01**
Last FU, median (IQR)			
Cr	0.98 (0.86–1.18)	0.99 (0.82–1.17)	.8
eGFR	82 (62–93)	76 (59–91)	.1
eGFR variation, median (IQR)˚	− 3 (− 9.1 to 3)	− 5 (− 12.9 to 1.2)	.1
Canalization, no. (%)			
> 3 days	9 (6)	14/188 (7.4)	.6
Ambulation, no. (%)			
> 2 days	4/148 (2.7)	7/183 (3.8)	.7
Pain at discharge, no. (%)			
> 2	7 (3)	5 (8)	.3
Pain management			
Paracetamol (g), median (IQR)	4 (3–6)	6 (4–8)	** < .001**
Additional NSAIDs, no. (%)	22/135 (16.3)	42/172 (24.4)	.09
Additional opioids, no. (%)	1/138 (0.7)	10/177 (5.6)	**.02**
LOS, days			
Median (IQR)	4 (4–5)	5 (4–6)	**.001**
90 days readmission, no. (%)	1/146 (0.7)	5/185 (2.7)	0.2

ccRCC, clear cell renal cell carcinoma; CD, Clavien-Dindo; Cr, serum creatinine; eGFR, estimated glomerular filtration rate; FU, follow-up; Hb, hemoglobin; LOS, length of stay; NSAID, non-steroidal anti-inflammatory drugs; POD, postoperative day. Student’s T test was used for continuous variables. Fisher's exact test was used for categorical variables.

^§^Hb drop is calculated as the difference between baseline and I POD.

˚eGFR variation is calculated as the difference between baseline and last follow-up.

Significant values are in bold.

**Table 4:** Multivariate logistic regression analysis of predictors of postoperative complications.

	Any grade postop complications	CD ≥ 3 postop complications p Value	OR (95% CI)	*p* Value
	OR (95% CI)			
Sex				
* Female*	(ref)		(ref)	
* Male*	0.79 (0.33–1.92)	0.6	0.61 (0.20–2.17)	0.1
Age	1.01 (0.98–1.05)	0.2	0.95 (0.87–1.03)	0.2
CCI	0.93 (0.69–1.25)	0.6	0.62 (0.28–1.35)	0.2
Tumor size	1.56 (0.98–1.85)	0.5	0.92 (0.85–1.05)	0.05
Clamp				
* No*	(ref)		(ref)	
* Yes*	0.91 (0.55–1.53)	0.1	1	
Ischemia time	1.04 (0.99–1.11)	0.1	0.95 (0.83–1.10)	0.5
Drain				
* No*	(ref)		(ref)	
* Yes*	0.79 (0.33–1.87)	0.5	3.62 (0.53–9.68)	0.9
EBL	1.11 (0.99–1.26)	0.1	1.57 (0.69–1.78)	0.6

## Discussion

The placement of a prophylactic drain after abdominal surgery represents an established clinical practice. It is based on surgeons’ habits, preferences, and empirical rather than scientific data. Literature evidence is controversial about the benefit derived from placing a drain. In the present study, we focused on the perioperative outcomes of RAPN to evaluate the impact of placing (or not) an abdominal drain at the end of the surgery.

In summary, we found a higher incidence of major postoperative complications in patients who had a drain placed. However, the insertion of an abdominal drain did not predict higher complications in multivariate analysis. The loss of statistical significance in multivariate analysis points out that the placement of a drain could be safely avoided after RAPN without increasing the risk of complications. Notably, the rate of tubeless procedures showed a statistically significant increase from 19% in the initial 2-year period assessed to 72% in the final 2-year period, potentially indicative of a combination of growing experience and confidence levels.

Our results are consistent with those reported by several comparative analyses available in the current literature. More than a decade ago, Abaza and Prall published a retrospective evaluation of a single surgeon case series suggesting that drain placement after RAPN could be routinely omitted without increasing the risk of complications^11^. A large multi-institutional retrospective study by Beksac et al. conducted on 904 patients undergoing transperitoneal RAPN showed no statistically significant difference in terms of overall/major postoperative complications and readmission rates. The authors concluded that drain can be omitted after RAPN, without detrimental effect on postoperative outcomes. Despite the large sample size, patients in the drain group had favorable characteristics compared to the “tubeless” patients, such as lower BMI and ASA score, and smaller and lower complexity masses. These aspects could have influenced the results observed^12^. Peyronnet et al. retrospectively compared 140 patients without abdominal drain insertion to 496 patients who had drain placed after RAPN. Again, adding a postoperative drain did not increase the risk of complications, whatever their grade. Nevertheless, the disparity in the sample size between the two cohorts may hamper the generalizability of these results^13^. Notwithstanding their limitations, both the studies agree on the safety of tubeless RAPN. Hence, the focus of the debate should be whether drain tubes represent a necessary diagnostic tool in the postoperative management of patients undergoing RAPN. Our results would suggest that clinicians may have other parameters upon which to base the decision-making process. Indeed, patients from the drain group had a significantly higher Hb drop which likely explained the higher rate of transfusions. However, this was not reflected by substantially different drain outputs. Moreover, bleeding events and rate of postoperative embolizations were comparable between the two groups, suggesting that conservative management may be sufficient in most cases. In this scenario, the diagnosis of a postoperative hemorrhagic complication relies on signs/symptoms and computed tomography scan, which remains the gold standard when suspecting postoperative complications^14^. For instance, Peyronnet et al. observed that the systematic placement of a prophylactic drain after RAPN did not decrease the number of postoperative computed tomography scans^13^. This could be extended to the detection of urinary fistulas as well, another reason for which a drain could be placed. Nevertheless, while patients in the drain group exhibited a statistically significant higher rate of collecting system violation and subsequent repair, the incidence of diagnosed urinary fistulas in our cohort remained extremely low (0.6%), in line with previously reported data^15^. Moreover, it has been suggested that postoperative drainage has some inherent limitations in diagnosing postoperative urinary fistula. Drain/serum creatinine ratio is referred to as the most reliable test for abdominal urinary leakage^16,17^. This diagnostic tool assumes that a violation of the collecting system will result in an increased ratio. However, an elevated drain/serum creatinine ratio does not necessarily indicate a urinary fistula, although a urinary fistula will invariably lead to an elevation of the ratio. Indeed, Williams et al. reported no statistically significant difference in terms of drain/serum creatinine ratio between patients with and without collecting system violation during RAPN^18^. Furthermore, the clinical significance of postoperative urinary leakages is yet to be defined since many urinary fistulae may resolve without needing any active treatment^5^. On the contrary, it has been suggested that drainage may harm abdominal fluid collection^19^.

We observed a significant difference in tumor features and intraoperative variables between the drain and no-drain groups. Patients in the former group had larger and more complex renal masses, leading to longer operative and ischemia time and higher blood loss despite the higher rate of on-clamp procedures. Kahn et al. performed a retrospective comparative analysis of patients who either underwent abdominal drain placement or did not, in which the drain cohort had unfavorable tumor and surgical features compared to the tubeless cohort. Authors reported a higher rate of major complications in the drain group, lacking statistical significance in multivariate analysis^20^. These results further confirm that more challenging procedures may incline the surgeon toward drain placement. However, this practice does not alter the postoperative course and serves to facilitate the diagnostic work-up of postoperative complications. The message we aim to convey is not to avoid abdominal drain placement per se, as certain renal masses and procedures will justify its use. It should be regarded as an additional tool tailored to specific surgical and patient needs, rather than a standardized component of the surgical procedure. In addition, the insertion of an abdominal drain after renal surgery could be double-edged. An abdominal drain itself could be related to postoperative complications such as hyperpyrexia, retained drain fragments, and patient discomfort, ultimately leading to prolonged hospital stay and reduced cost-effectiveness^21,22^. Our results sustain this hypothesis since the present analysis highlighted a statistically significant longer hospitalization in the drain group. We sought to evaluate patient discomfort by analyzing different patient-related outcomes. While no differences were observed in terms of time to canalization (*p* = 0.6), time to deambulation (*p* = 0.7), and pain at discharge (*p* = 0.3), the drain cohort exhibited a statistically significant higher need for paracetamol (*p* < 0.001) and additional opioids (*p* = 0.02). These results may cautiously be attributed to the presence of an abdominal drain, as reported in other studies analyzing postoperative pain in patients with drain placement. In a randomized controlled trial by Kriegmair et al., patients undergoing drain insertion after open partial nephrectomy experienced higher post-operative pain, and delayed mobilization than those without a drain^5^. We must admit that drain placement for open partials involves a large incision below a flank, which is likely to be more painful. We are not entirely certain that this is comparable to drain placement after RAPN, which typically exits through a small pre-existing port site incision. Nevertheless, these findings gain particular significance when considering the attention currently given to a faster recovery and shorter hospital stay, further emphasizing the concept of minimal invasiveness of surgical procedures. Efforts in this direction have been made by developing ERAS protocol for urologic surgery^23^. Nevertheless, these regimens are underutilized in urological practice due to barriers associated with implementing optimal strategies for intravenous fluid provision, antibiotic therapy, and pain management^24^. Therefore, identifying preventable and avoidable risk factors that may affect a seamless postoperative course, such as using abdominal drainage, is crucial in properly managing patients undergoing RAPN.

The present study has limitations that must be acknowledged. First, the retrospective study design carries inherent bias despite the prospective granular data collection that only partially mitigates the impact of confounders. Second, the study was conducted in a high-volume tertiary referral center, potentially limiting the generalizability of its findings. Last, we could not address postoperative complications specifically related to drainage, which is essential for evaluating the potential harm associated with abdominal drain insertion. However, the heterogeneity of these complications, along with difficulties in their definition, hampers objective data collection and analysis.

On the other hand, the study benefits from a large sample size of consecutive patients with adequate follow-up, augmenting our results' validity and applicability. Moreover, it relies on a comprehensive data collection process to address a debated and uncertain aspect in optimizing the postoperative course of patients undergoing RAPN. In addition, we believe that including patient-related outcomes strengthens the present study, adding evidence to the existing literature, primarily based on retrospective studies often lacking a specific analysis of these outcomes.

Despite the promising findings from our analysis, we recognize that there are circumstances where surgeons may view the placement of a drain as a prudent precaution, especially when there are concerns regarding postoperative complications. Drains have the potential to facilitate early detection and intervention in case of complications following surgery. While advancements in RAPN techniques have led to reduced complication rates, we believe that proactive measures like drain placement can still play a role in optimizing postoperative care and outcomes, particularly as we extend the indications for nephron-sparing surgery to more complex cases.

## Conclusions

The present findings suggest the safety of avoiding abdominal drain placement, given the comparable likelihood of facing any grade and major postoperative complications. A relationship between drain insertion and increased postoperative pain and the need for opioids was observed, potentially influencing patient comfort and recovery. Within the limitations related to the retrospective study design, our results confirm the safety of avoiding prophylactic drain placement and the need for personalized decision-making, with careful consideration of patient and procedural factors.

## Data Availability

The datasets used and/or analyzed during the current study are available from the corresponding author on reasonable request.
